# Biliary Epithelial Senescence and Plasticity in Acute Cellular Rejection

**DOI:** 10.1111/ajt.12271

**Published:** 2013-06-10

**Authors:** J G Brain, H Robertson, E Thompson, E H Humphreys, A Gardner, T A Booth, D E J Jones, S C Afford, T von Zglinicki, A D Burt, J A Kirby

**Affiliations:** 1 Institute of Cellular Medicine, Newcastle UniversityNewcastle upon Tyne, UK; 2 Centre for Liver Research, School of Infection and Immunity University of BirminghamBirmingham, UK; 3 NIHR BRU Queen Elizabeth Hospital BirminghamUK; 4 Institute for Ageing and Health, Newcastle UniversityNewcastle upon Tyne, UK; 5 Clinical Deanery, Newcastle UniversityNewcastle upon Tyne, UK

**Keywords:** Liver, epithelial de-differentiation, biliary disease, allograft rejection

## Abstract

Biliary epithelial cells (BEC) are important targets in some liver diseases, including acute allograft rejection. Although some injured BEC die, many can survive in function compromised states of senescence or phenotypic de-differentiation. This study was performed to examine changes in the phenotype of BEC during acute liver allograft rejection and the mechanism driving these changes. Liver allograft sections showed a positive correlation (p < 0.0013) between increasing T cell mediated acute rejection and the number of BEC expressing the senescence marker p21^WAF1/Cip^ or the mesenchymal marker S100A4. This was modeled *in vitro* by examination of primary or immortalized BEC after acute oxidative stress. During the first 48 h, the expression of p21^WAF1/Cip^ was increased transiently before returning to baseline. After this time BEC showed increased expression of mesenchymal proteins with a decrease in epithelial markers. Analysis of TGF-β expression at mRNA and protein levels also showed a rapid increase in TGF-β2 (p < 0.006) following oxidative stress. The epithelial de-differentiation observed *in vitro* was abrogated by pharmacological blockade of the ALK-5 component of the TGF-β receptor. These data suggest that stress induced production of TGF-β2 by BEC can modify liver allograft function by enhancing the de-differentiation of local epithelial cells.

## Introduction

The rate of acute cellular rejection in liver allografts has fallen from 60–70% to 10–20% of graft recipients and classical ductopaenic rejection has all but disappeared from clinical practice 1. However, this reduction has been accompanied by an increase in factors limiting the quality and length of life of liver transplant recipients 1–3. There are significant sequelae associated with the administration of immunosuppressive agents, stimulating interest in regimes designed to induce tolerance to liver grafts and the ability to maintain grafts with minimal or no immunosuppression 4. The limited evidence available also points to increased fibrosis in patients classed as tolerant, with no evidence of either acute or chronic rejection 4. There is also interest in other phenomena associated with graft immune responses, such as nonclassical antibody-mediated rejection and late acute cellular rejection 5,6.

While fibrosis is arguably a reversible endpoint, there has been little study of the factors determining transition from acute to chronic disease. Unlike other transplanted organs, there is evidence that patients suffering an episode of acute liver allograft rejection have a favorable outcome in terms of survival with a functioning graft 7, raising the possibility that acute rejection can have beneficial effects on the allograft immune infiltrate. Effects of biliary injury in this setting have not been studied; however, there has been recent interest in the response of the biliary epithelium during the development of ductopaenic liver disease, including chronic rejection 8–10. Lethally injured cells typically undergo either necrosis or apoptosis. However, sublethal injury can lead to a number of conserved processes, including senescence 10 or autophagy 11. Cellular senescence is defined as loss of the ability to divide and can be induced by replication (exhaustive or replicative senescence) or toxic effects, for example, from oncogenes or chemokines, and is well defined in chronic ductopaenic rejection 12. In practical terms, defining senescence relies on the detection of phenotypic markers as it is not possible to follow the life cycle of cells *in vivo*. The functional definition of senescence could therefore be described as cells exhibiting markers, at the time of sampling, which indicate further division is unlikely. There are no reports describing senescence in acute cellular rejection of liver allografts.

Nakanuma and coworkers 10,13 have investigated the effect of senescence on biliary epithelial cells (BEC) in chronic biliary disease, demonstrating a correlation between number of senescent BEC and the progression of a number of diseases in human biopsies 8,10,13. In replicating the biopsy features *in vitro*, Sasaki et al. 14 used oxidative stress to induce murine biliary epithelial senescence. Exposure to hydrogen peroxide replicated the cell expression of the senescence marker p21^WAF1/Cip1^ observed *in vivo*. Furthermore, this model system permitted investigation of chemokine secretion as part of the senescence associated secretory phenotype (SASP) 15. The chemotactic effect may lead to the recruitment of further immune cells and therefore progression of disease by increased necroinflammation. This represents a shift in thinking from the epithelium as a passive recipient of injury to a potential effecter in immunologically mediated disease.

While these data are interesting, it remains unclear whether such bile duct responses occur only in the setting of persistent chronic inflammatory disease or whether they are also seen early in response to BEC injury, as implied by Nakanuma's group 16. While the ability of senescent BEC to induce chemotaxis of immune cells and stellate cells has been assessed, there has been no conclusive proof of a direct mechanism by which senescence of these cells may lead from acute to chronic disease or an assessment of the fibrogenic potential of senescent cells.

This study was designed to evaluate the presence of senescent BEC in acute cellular rejection by evaluating the association between senescent biliary cells and the grade of acute cellular rejection in transplant biopsies. To investigate the possible underlying mechanism linking acute injury to disease severity and possible progression, the potential of senescent biliary epithelium to secrete pro-apoptotic, profibrogenic, and immunomodulatory factors in the SASP was then explored using a model system *in vitro*.

## Materials and Methods

### Ethics

Approval was obtained from a local regional ethics committee for the use of anonymized patient samples (approval reference: REC 06/Q0905/150). All patients were treated on a standard algorithm receiving prednisolone, tacrolimus, and azathioprine. All biopsies were taken before escalation of therapy.

### Immunohistochemical (IHC) triple staining of human liver biopsies

Paraffin embedded tissue sections were dewaxed and rehydrated. Antigen retrieval was followed by blocking and peroxide incubation to quench endogenous peroxidase activity. Primary antibody was then added (anti-p21^WAF1/Cip1^ (Santa Cruz, Santa Cruz, TX) 1:50 or anti-CD3 (DAKO, Cambridge, UK) 1:100) or Ki67 (Immunotech, Monrovia, CA) followed by biotinylated goat anti-mouse IgG (Vector, 1:200) and developed with Nickel 3,3′ diaminobenzidine tetrahydochloride (NiDAB). A serial incubation with anti-Cytokeratin 19 (DAKO; 1:1000) and anti S100A4 (BD, 1:200) followed with subsequent addition of peroxidase conjugated horse anti-mouse IgG (Vector; 1:100; developed with 3, 3′ diaminobenzidine tetrahydochloride (DAB)) and biotinylated goat anti-rabbit IgG (Vector; 1:200; developed with the Vector ABC-AP kit). Sections were counterstained, dehydrated, and mounted with DPX. Dual and single stains were performed in a similar manner, with inclusion of the γH2AX antibody (Cell Signal, Danvers, MA).

### Analysis

The cases represented 9 ‘time zero’ biopsies (tissue taken at the point of intraoperative reperfusion of a transplanted liver) as control tissue and 25 biopsies of transplanted liver with varying grades of acute rejection (mild, moderate and severe, with all biopsies taken before augmentation of immunosuppression). The sections were scanned using an Aperio slide scanner at 20× and remotely accessed files were analyzed. Each biopsy was assessed by two observers (J.G.B. and H.R.) and scored for number of portal tracts, number of bile duct radicles, maximum number of bile ducts per portal tract, number of p21^WAF1/Cip1^ positive BEC, number of S100A4 BEC, number of dual stained BEC (S100A4 and p21^WAF1/Cip1^), and number of p21^WAF1/Cip1^ and S100A4 BEC adjacent to one another. There was good agreement between the observers, any discrepancies were noted and a consensus opinion reached. As a number of immune cells stain positive for S100A4, there was a chance that infiltrating macrophages or T cells could be mistaken for BEC. This was usually straightforward to assess morphologically. For control experiments 14 separate cases (five time zero and nine acute cellular rejection) were stained with Ki67/S100A4/CK19 to act as a proliferation marker control group. A further eight cases of posttransplant biliary anastomotic injury/stricture were stained with p21^WAF1/Cip1^/S100A4/CK19 as positive control tissue. To further support the presence of senescent cells 15 cases (10 ACR and five time zero) were labelled with antibodies specific for Ki67 and γH2AX.

### Cell culture

The immortalized H69 cell line was created by, and obtained from, Grubman 17 from human intrahepatic BEC. These cells exhibit characteristics of normal human biliary epithelium, with expression of CK7 and 19. The H69 cells were cultured as described 17.

Primary biliary epithelial cells were isolated from human explanted livers and cultured on plasticware coated with rat tail collagen as described 18.

### Immunofluorescence and densitometry

For immunofluorescence experiments, immortalized BEC were seeded onto glass eight-chamber slides (Becton Dickinson, Cowley, UK) at 50 000 cells per chamber with 250 µL of culture medium. Cells were cultured for 24–48 h until a confluent monolayer was formed. Following optimization, cells were subjected to oxidative stress by the addition 200 µM H_2_O_2_ (final concentration) for 2 h followed by washing with PBS and addition of fresh culture medium. After 24, 48, 72, 96, or 120 h the cells were fixed with 4% phosphate-buffered paraformaldehyde and permeabilized with 0.1% Triton X-100. The cells were then blocked with 5% BSA.

Primary antibodies specific for S100A4 (DAKO, 1:100), αSMA (Sigma, Poole, UK, 1:100), E-cadherin (BD Biosciences, Oxford, UK 1:50), ZO-1 (Invitrogen, Grand Island, NY, 1:100), p21^WAF1/Cip1^ (Santa Cruz, 1:100), fibronectin (Sigma, 1:100) or vimentin (DAKO, 1:100) were added in 5% BSA. For each time point and antigen a no-primary antibody treated preparation and a nonspecific isotype/species specific primary antibody preparation were used as negative controls. The cells were then washed with 0.1% Tween-20 in PBS prior to addition of FITC-conjugated anti-mouse or anti-rabbit (DAKO, 1:100) secondary antibody in 5% BSA. Following washing, slides were stained with DAPI (at a concentration of 1 µg/mL). Slides were then washed in PBS and mounted in fluorescence mounting medium (DAKO). Storage was at 4°C.

Visualization was by immunofluorescence using a Leica TCS SP2 UV laser-scanning confocal microscope (LSCM) to detect FITC (excitation 488 nm, emission 510–535 nm) and DAPI (excitation peak 360 nm, emission 450–550 nm). Emission levels were set against a control section. Representative z-series were taken of each section/specimen and used for densitometric and morphological analysis. Densitometry analysis was by ImageJ software. All experiments were conducted in triplicate.

### Western blotting

Immortalized BEC growing in 75 cm^2^ flasks were washed and 100 µL of lysis buffer (Phosphosafe™ Merck KGaA, Darmstadt, Germany) added. Total protein concentration was estimated by BCA protein assay kit (Pierce, Rockford, IL, USA). This allowed production of a standard curve for absorbance at 562 nm. Extracts were stored at −20°C. For sodium-dodecyl sulphate–polyacrylamide gel electrophoresis (SDS–PAGE), samples were supplemented with 10% β-mercaptoethanol and 4× NuPAGE sample buffer (Invitrogen), before boiling, cooling, and addition to SDS–PAGE gels.

Twenty micrograms of total protein lysate per well was separated by 4–12% gradient SDS–PAGE. Separation was carried out using stable voltage, (20 V per gel). Separated proteins were transferred from the gel to Hybond-P nitrocellulose membrane (Amersham Pharmacia Biotech, Little Chalfont, UK) overnight at 50 mA. Nonspecific antibody binding sites on the membrane were blocked with 5% skimmed milk in triethanolamine-buffered saline (TBS). Primary antibodies were diluted in 5% milk/TBS to optimal concentrations. Membranes were incubated with primary antibodies overnight before washing in TBS supplemented with 0.1% Tween-20. Membranes were then incubated with secondary antibodies conjugated with HRP (horse radish peroxidase). After washing, the membrane was developed using Pierce Pico Chemiluminescence kit (Pierce, Rockford, IL). Visualization was by use of G-Box CCD camera (Syngene, Cambridge, UK).

Following development, membranes were stripped of antibodies with stripping buffer (62.5 M Tris, 2% SDS, 100 mM β-mercaptoethanol, pH 6.8). Membranes remained in the cooling buffer with agitation for a further 30 min. Membranes were washed in TTBS, reblocked in 5% skimmed milk, and incubated overnight with anti-β-tubulin antibody (Sigma, 1:5000) in 5% skimmed milk and TBS. Secondary antibody was anti-rabbit HRP conjugated antibodies (Sigma, 1:5000) with development as for primary antibodies. The intensity of β-tubulin was used to validate equal loading.

### ELISA

ELISA kits specific for the TGF-β1 and TGF-β2 isoforms were purchased from R&D Systems (UK). Cell culture medium from two separate time courses was retained for analysis. This was treated with acid (50 µL 6 M HCl per mL medium for 10 min followed by neutralization with 50µL per mL of 6 M NaOH) in order to assess total rather than the active amount of TGF-β. The kits were used in accordance with the manufacturer's instructions.

### Real-time quantitative polymerase chain reaction (qPCR)

RNA isolation was performed according to the method developed by Chomczynski and Sacchi 19. TRIzol reagent (Sigma) was used according to the manufacturer's instructions. RNA was quantified and quality assessed using a nanodrop spectrophotometer (NanoDrop ND-1000, Thermo Scientific, Wilmington, DE). Isolated RNA was reverse transcribed to cDNA using the AffinityScript Multi Temperate cDNA synthesis kit (Agilent Technologies, Santa Clara, CA) according to the manufacturer's instructions.

The qPCR experiments in this project utilized TaqMan (Applied Biosystems, Paisley, UK) chemistry and were performed in a MicroAmp Optical 96 well plate (Applied Biosystems) on a StepOnePlus real-time PCR machine (Applied Biosystems) with each well containing 1 µL TaqMan primer-probe (all primers were exon-spanning), 1 µL cDNA, 10 µL Mastermix and 8 µL RNase free water. Triplicate technical replicates were performed for each experiment with GAPDH serving as the housekeeping control.

### Statistical analysis

Statistical analysis was performed using Prism software version 5.0 (Graph Pad Software, San Diego, CA). Parametric data were assessed by Student's t-test or one way ANOVA. Statistical significance was defined as a p-value of 0.05 or less. All error bars represent the standard error of the mean (SEM). Nonparametric data were analyzed by the Kruskall–Wallis test.

## Results

### Senescence and plasticity markers in human liver transplant biopsies

**Figure 1:**
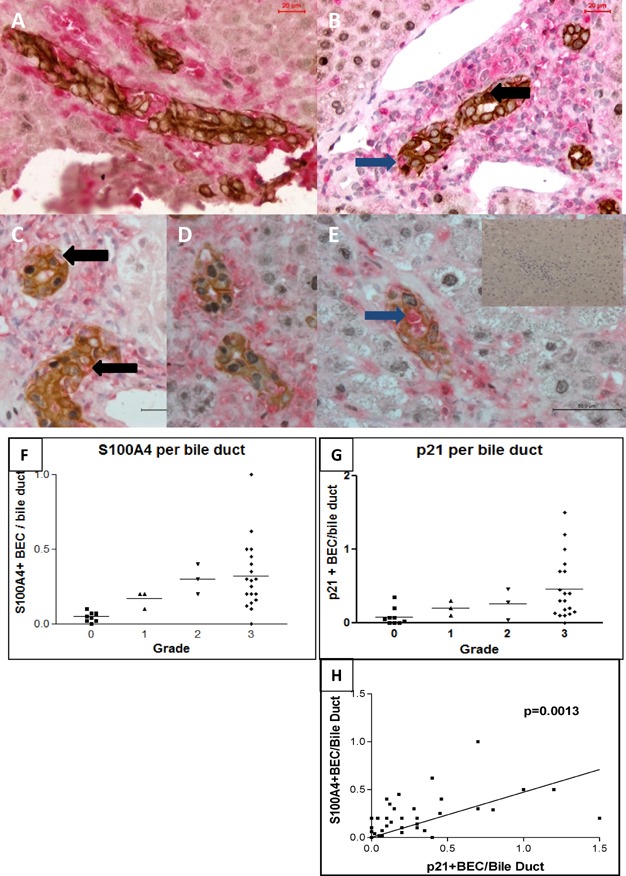
Triple color immunohistochemistry of senescence and plasticity markers in human liver transplant biopsies Panels A–E represent photomicrographs depicting the relative staining attained using the three color technique. Brown staining reflects CK19 expression, Black p21^WAF1/Cip^ and red S100A4. Cells staining dual positive for S100A4 and CK19 are shown with black arrows. Infiltrating cells (blue arrow) were excluded morphologically or with reference to further immunohistochemistry. Panel A represents a time zero biopsy, used as control. Panels B and C mild acute cellular rejection and panels D and E moderate and severe ACR, respectively. Inset image shows section stained with no primary antibody. Panels F–H show graphs attained by scoring the biopsies. Panel F indicates the number of S100A4 positive biliary epithelial cells per bile duct, with a clear increase with increasing grade of acute cellular rejection. Panel G indicates the same association with S100A4 p21^WAF1/Cip^ expression and Panel H shows the correlation between S100A4 and p21 expression.

Triple color immunohistochemistry of transplant biopsies and time zero controls were scored and are shown in Figure 1, panels A–E. S100A4 cells (red) are clearly delineated, as is the strong nuclear positivity of p21^WAF1/Cip^ (black), allowing co-localization with the BEC specific CK19 (brown). p21^WAF1/Cip^ positive cells indicated senescence and those positive for S100A4 indicated de-differentiation/presence of cellular plasticity. During assessment, dual positive S100A4 and p21^WAF1/Cip1^ positive biliary epithelial cells were not seen. Also, there were few examples of adjacent p21^WAF1/Cip1^ and S100A4 positive BEC. Blue arrows indicate infiltrating S100A4 positive cells, black arrows show BEC stained with either S100A4 or p21.

Analysis showed significant increase of p21^WAF1/Cip1^ (p = 0.034) and S100A4 (p = 0.0068) positivity per bile duct with increasing grades of acute cellular rejection (Figure 1, panels F, G). Using the Spearman correlation test it was demonstrated that there was a statistically significant correlation (p = 0.0013) between the expression of S100A4 and p21^WAF1/Cip1^ (Figure 1, panel H).

**Figure 2:**
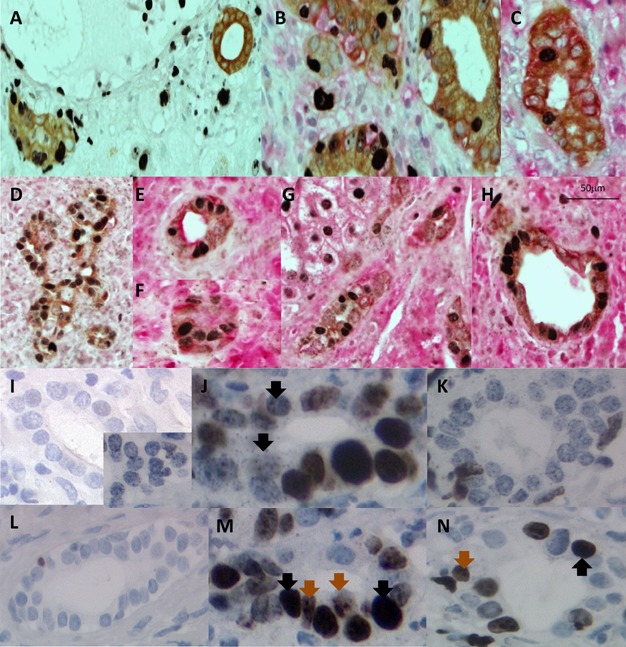
Triple color immunohistochemistry of proliferation markers and control human transplant biopsies Panels A–C represent transplant biopsies stained for Ki67 (black), S100A4 (red) and CK19 (brown). Panels A demonstrates mild rejection and Ki67 positive BEC are clearly seen in the absence of S100A4. Panel B shows Ki67 and S100A4 dual positive BEC in moderate rejection, with the spatial relationship between single positive S100A4 or Ki67 positive BEC apparent. Panel C shows a field from a severe rejection biopsy, indicating the number of S100A4 positive BEC relative to Ki67 positive BEC. Panels D–H represent fields from human liver transplant recipients with biliary anastomosis obstruction or stricturing. These are stained with p21^WAF1/Cip^ (black), S100A4 (red), and Ck19 (brown) as control material. The abundance of staining for all antigens is apparent. Panels I–K show dual staining for Ki67 (brown) and γH2AX (black) Panel I shows ACR biopsy material, the main image is a negative control, the inset image is a single stained γH2AX, showing the characteristic punctate nuclear staining pattern. Panel J is from a moderate ACR biopsy, clear Ki67 negative and γH2AX positive cells are seen (arrows). Panel K shows a severe ACR biopsy with a clear increase in the numbers of Ki67 negative and γH2AX positive cells. Panels L–N show dual stained Ki67 (brown, brown arrows) and p21^WAF1/Cip1^ (black, black arrows). Panel L is from a mild ACR showing only Ki67 positivity, Panel M is moderate and panel N severe ACR.

As previously described 20, p21^WAF1/Cip1^ alone is not a specific marker for senescence, though it is much more specific when described in Ki67 negative cells. In line with the published literature 20,21, the presence of Ki67 negative/γH2AX positive cells is regarded as the gold standard for senescence detection. To define whether there was further evidence of senescence in ACR, biopsies were also stained for Ki67 and γH2AX. γH2AX positivity was defined by the presence of more than five positive foci per nucleus. Cells positive for γH2AX (and negative for Ki67) were seen with increasing frequency (Figure 2 panels I–K) in cases with increasing ACR grade. For completeness dual staining of Ki67 and p21^WAF1/Cip1^ was performed (Figure 2 panels L–N). This showed that Ki67 negative p21^WAF1/Cip1^ positive cells were present. Enhanced production of reactive oxygen species (ROS) is another component of the senescent phenotype, leading to accumulation of autofluorescent, lipofuscin-like material 34. BEC in ACR show greatly enhanced autofluorescence (Figure S3).

### Proliferation and plasticity markers in human liver transplant biopsies

Triple color immunohistochemistry was performed for the markers Ki67 (black), S100A4 (red), and CK19 (brown) (Figure 2 panels A–C). As previously reported in the literature, there was an increase in the Ki67 positive cells with increasing grade of rejection. 80–85% of the Ki67 positive cells in moderate or severe rejection were also co-stained with S100A4; those that were not co-stained were adjacent to S100A4 positive BEC. The majority of S100A4 positive BEC were not dual stained for Ki67 as shown in panel C. Figure 2 panels D–H show images of biopsies from transplant recipients with biliary anastomotic stenosis or obstruction. Stains are p21^WAF1/Cip^ (black), S100A4 (red), and CK19 (brown).

### Oxidative stress-induced senescence in BEC cultures

Following treatment by H_2_O_2_ immortalized BEC were assessed for senescence by detection of nuclear p21^WAF1/Cip1^ expression as this is related to cell cycle arrest and senescence 22. The numbers of positive nuclei per high power field (20× lens; LSCM) were counted, with one field from each of the triplicate of experiments included in the analysis. Treatment with H_2_O_2_ at 200 µM for 2 h produced significant nuclear expression of p21^WAF1/Cip1^ after 48 or 72 h, consistent with the literature.

**Figure 3:**
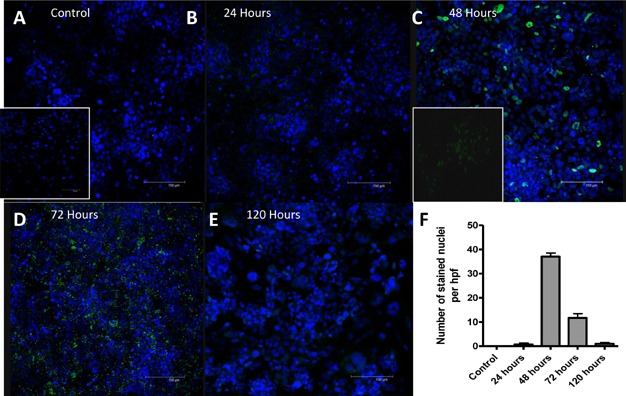
Photomicrographs demonstrating p21^WAF1/Cip^ expression The nuclei of the Immortalized BEC are visualized with DAPI and are blue. p21^WAF1/Cip^ is stained with FITC and is green. Panels A, B, C, D, and E show results observed at time 0, 24, 48, 72, and 120 h, respectively. The increase in nuclear p21^WAF1/Cip^ staining is seen with a peak at 48 h. Staining seen at 72 h is largely cytoplasmic, indicating phosphorylated and therefore inactive p21^WAF1/Cip^. The nuclear staining is shown graphically in panel F, three representative fields from three separate experiments at each time point. Primary BEC were also stained, images are shown inset for control (inset A) and at 48 h (inset C).

Peak p21^WAF1/Cip1^ expression was after 48 h, with a rapid decline after this time (Figure 3 panels A–E). This was assessed by one way ANOVA p < 0.0001. Post hoc t-tests showed that control versus 48 and 72 h were significant at p < 0.001. The rises in expression between 24, 48, and 72 h were all significant at p < 0.001. Comparable findings were demonstrated using primary BEC, where untreated cells showed no expression of p21^WAF1/Cip1^, 200µM hydrogen peroxide gave a rapid up-regulation in nuclear p21^WAF1/Cip1^ at 48 h with down-regulation after 72 h (inset images Figure 3 panels A and C).

### Oxidative stress-induced epithelial de-differentiation in BEC cultures

**Figure 4:**
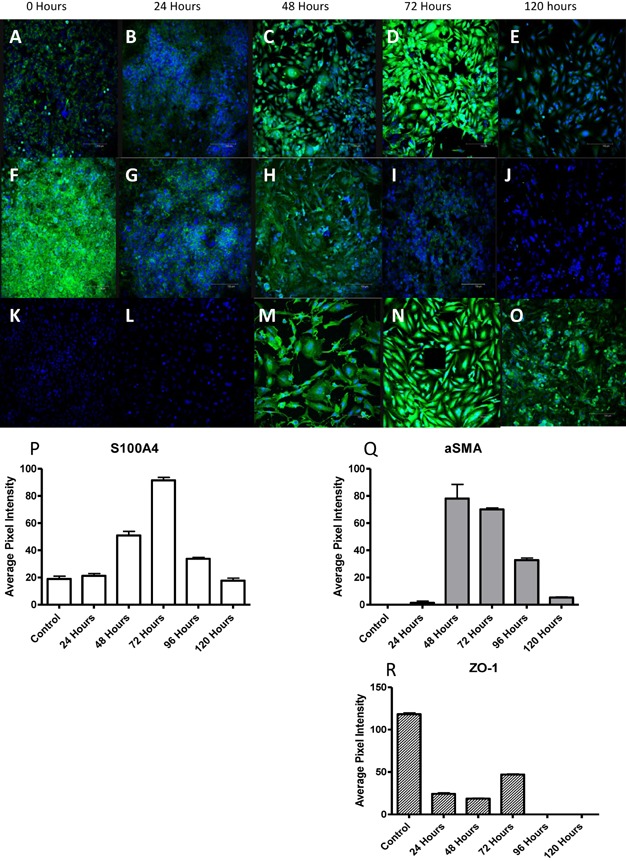
Photomicrographs depicting change in antigen expression over time Each photomicrograph is a representative sample from triplicate experiments. The incubation period post peroxide exposure is noted along the top. Panels A–E show the change in S100A4 expression, F–J the change in ZO-1 expression and K–O the change in α-SMA expression. Significant changes in morphology can be seen, with spindle cell morphology apparent after 48 h. For each of the antigens ZO-1, α-SMA and S100A4, densitometric analysis was performed as described in methods. The results are tabulated in panels P–R, based on triplicate readings with the mean and standard error shown. Clear changes in expression of the antigens are seen, with increasing S100A4 and α-SMA and decreasing ZO-1 expression statistically significant (one way ANOVA) at p = 0.0096 (S100A4), α-SMA at p = 0.0068 and ZO-1 at 0.0087.

Immortalized BEC showed a morphological change 72 h after exposure to H_2_O_2_ from typical cobblestone to spindle cell morphology with increased expression of S100A4, a marker of de-differentiation (Figure 4 panels A–E). This morphology was consistent with the de-differentiation seen when immortalized BEC cells are exposed to TGF-β1 23. Triplicate cultures of immortalized BEC were exposed to 200 µM H_2_O_2_ for 2 h followed by incubation for 24, 48, 72, 96 or 120 h. To control for potential tissue culture induced changes, triplicate cultures were plated and incubated for the same length of time as the treated cultures, with no change in morphology or expression. At all time-points, cultures were stained for αSMA (Figure 4 panels K–O), S100A4 (Figure 4 panels A–E), fibronectin, Vimentin, and ZO-1 (Figure 4 panels F–J). In all cases, the secondary antibody was FITC-conjugated (green fluorescence, Figure 4). Densitometric analysis was performed to quantify changes in antigen expression. This involved assessment of a defined area of each Z series obtained, one field from each of the three cultures. These data are presented in Figure 4 panels P–R. For each antigen a one-way ANOVA was performed, showing statistical difference in the expression of S100A4 (p = 0.0096), αSMA (p = 0.0348) and ZO-1 (p = 0.0018). Post hoc t-tests demonstrated that αSMA expression was significantly increased at 48 h compared to control and also increased between 24 and 48 h. ZO-1 expression was significantly decreased at 24, 48 and 120 h versus control.

**Figure 5:**
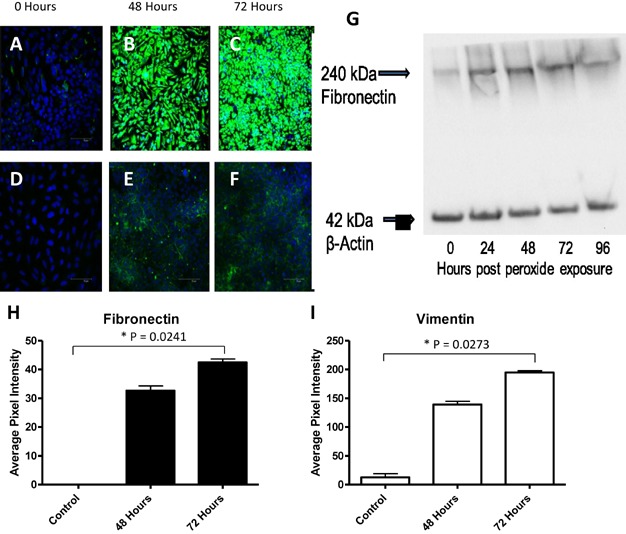
Extracellular matrix expression The expression of extracellular matrix components was assessed by fluorescence microscopy and confirmed by western blot. The expression of Vimentin (panels A–C) and fibronectin (panels D–F) is clearly absent at baseline but is apparent at 48 and 72 h post peroxide exposure. The fluorescence was quantified by densitometry and data shown in panels H and I (significant after 72 h at p = 0.0273 and p = 0.0241, respectively). Cell lysates from all time points are shown, following separation by SDS–PAGE (panel G), and also show a significant increase in fibronectin expression.

Further data from the time points of maximum expression of αSMA and S100A4 (48 and 72 h) showed an increase in expression of ECM components fibronectin (p = 0.0176) and Vimentin (p < 0.0001) (Figure 5 panels A–F, H and I). In order to provide further support for this change in antigen expression, cell lysates were separated by SDS–PAGE and the membranes probed using the same antibodies. These showed expression of fibronectin (at correct molecular weight, Figure 5 panel G) that increased from baseline, peaking at 48–72 h. Following all other experiments the cells that had been passaged were stained for the epithelial markers in order to verify that the immortalized BEC cell lines had retained their epithelial characteristics. These cells exhibited strong baseline staining for CK19 and E-cadherin (Supplementary Figure S1).

### Oxidative stress-induced de-differentiation of cultured BEC is TGF-β dependent

**Figure 6:**
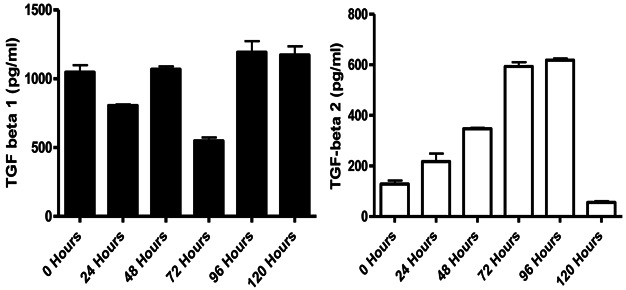
TGF-β ELISA Two ELISAs were performed to assess total TGF-β expression. The level of TGF-β1 (panel A) appears largely constant but the expression of TGF-β2 (panel B) shows almost a fourfold increase between time 0 and 72 h (p = 0.0058), where it appears to plateau before falling sharply.

To determine whether the observed de-differentiation in immortalized BEC was due TGF-β signaling, cell culture medium from peroxide treated immortalized BEC was retained to perform TGF-β ELISA. Following acid treatment to permit total TGF-β to be detected, these media were assayed in triplicate against controls. These data demonstrated TGF-β1 levels to be not significantly raised; whereas TGF-β2 levels were raised significantly over time (p = 0.006, Figure 6).

**Figure 7:**
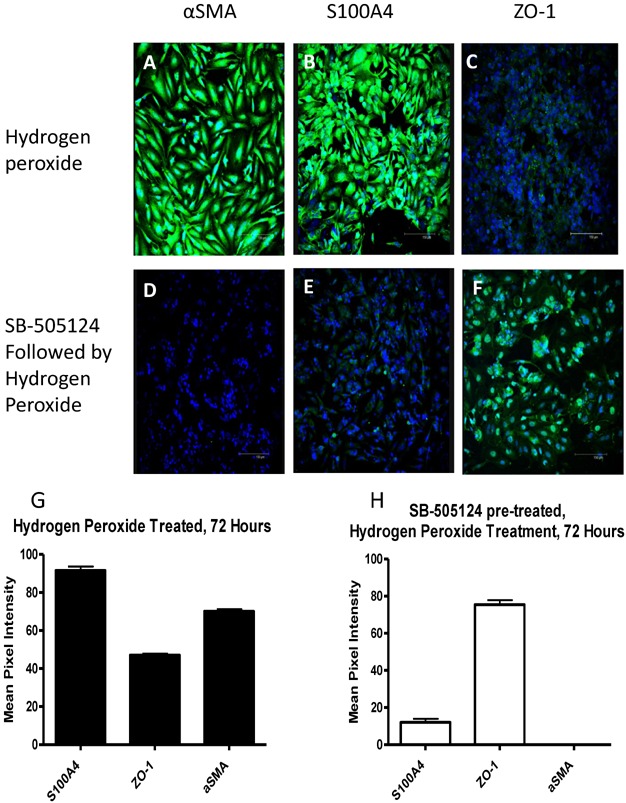
TGF-β receptor blockade Panels A–C show antigen expression at 72 h post H_2_O_2_ exposure as seen in Figure 3. Panels D–F show antigen expression of immortalized BEC cells that were treated with the ALK-5 inhibitor SB-505124 at 1µM for 1 h before H_2_O_2_ exposure and incubation for 72 h; no induction of αSMA or S100A4 was observed while ZO-1 expression was retained. Densitometric analysis is presented in panels G and H; two way ANOVA shows statistical significance at p < 0.0001.

In order to demonstrate causality in this *in vitro* system, a triplicate culture of immortalized BEC was incubated with a TGF-β type 1 receptor serine/threonine kinase (ALK5) inhibitor (SB-505124) at an optimal concentration of 1 µM (data not shown), for 1 h before exposure to 200 µM H_2_O_2_ for 2 h followed by incubation for a further 72 h before staining for the antigens S100A4, ZO-1, p21 and αSMA (Figure 7 panels A–F). Densitometric analysis was performed as above (Figure 7 panels G and H). The increased expression of S100A4 and αSMA and the decrease in ZO-1 normally produced by H_2_O_2_ exposure were almost completely abrogated by inhibition of TGF-β signaling. Statistical analysis between the SB-505124 treated and untreated, H_2_O_2_ exposed groups at 72 h, by two-way ANOVA was positive at p < 0.0001.

### TGF-β and p21^WAF^^1/^^C^^ip1^ RNA expression in BEC cultures

**Figure 8:**
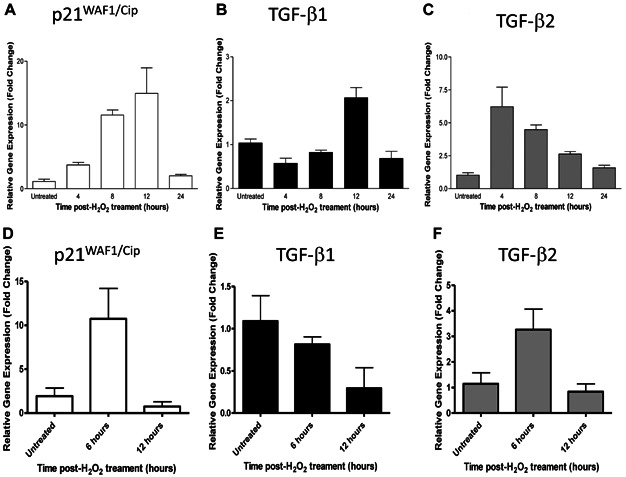
qPCR of immortalized and primary BEC Graphs shown represent triplicate replicates of representative qPCR experiments. Panels A–C show expression of mRNA in immortalized BEC cells at time points after exposure to 200 µM H_2_O_2_. Panels D–F show expression of mRNA in primary BEC at time points after 200 µM H_2_O_2_ exposure. One way ANOVA showed p21^WAF1/Cip^ expression to be significant at p < 0.001 (immortalized BEC) and p = 0.0285 (primary BEC) and TGF-β2 expression to be significant at p < 0.001 (immortalized BEC) and p = 0.042 (primary BEC).

Both immortalized and primary BEC were assessed for the expression of mRNA encoding p21^WAF1/Cip1^, TGF-β1 and TGF-β2. Figure 8 demonstrates a clear increase in p21^WAF1/Cip1^ expression (panel A) peaking at 12 h after treatment with H_2_O_2_ before falling back to baseline by 24 h. TGF-β1 showed a slight increase at 12 h whereas TGF-β 2 showed a significant sixfold increase (Figure 8 panel C) at 4 h which was sustained, falling back to near baseline by 24 h. To determine if the observed expression of p21^WAF1/Cip1^ in primary BEC was also associated with TGF-β expression, primary BEC were either untreated or treated with 200 µM hydrogen peroxide and incubated for 6 or 12 h from initial exposure and treated as the immortalized BEC. The primary cells also showed an increase in p21^WAF1/Cip1^ (Figure 8 panel D) and TGF-β2 expression (Figure 8 panel F) at 6 h followed by a fall back to baseline by 12 h. There was only a decrease in TGF-β1 expression (Figure 8 panel E).

### Activation of TGF-β by BEC

In order to assess the potential of BEC to activate latent TGF-β, immortalized BEC cultures were assessed for their ability to up regulate β6 integrin. Immortalized BEC were either used without treatment or following 200 µM H_2_O_2_ for 2 h and incubation for 24 or 48 h. At baseline expression of β6 integrin was not present; there was a significant up regulation in response to oxidative stress (p < 0.001; Supplementary Figure S2).

## Discussion

This study suggests that cellular senescence plays a key role in acute cellular rejection of liver allografts. A clear positive correlation was demonstrated between the number of p21^WAF1/Cip1^ positive biliary epithelial cells and increasing grade of acute rejection. This is consistent with data from other groups detailing severity in chronic cholestatic liver diseases 10,13,16. In addition to its role as a marker of senescence, p21^WAF1/Cip1^ can also be upregulated during the G1 phase of the cell cycle 24. Ki67 is a marker of proliferation present throughout all active phases of the cell cycle 25. In order to differentiate senescent and proliferating cells, Ki67 was used in a triple label in place of p21^WAF1/Cip1^. This demonstrated that Ki67 and p21^WAF1/Cip1^ positive BEC occupy different cellular niches. Whereas p21^WAF1/Cip1^ positive cells never co-stained for S100A4 and were never seen adjacent to S100A4 stained BEC, Ki67 stained cells were always either co-stained for S100A4 or adjacent to S100A4 positive BEC in moderate to severe rejection (though in mild rejection Ki67 positive cells were seen in the absence of S100A4). These results are compatible with a model in which the BEC that express p21^WAF1/Cip1^ are nonproliferating, senescent cells while other BEC, which may also express S100A4, are actively proliferating. The presence of the senescence marker γH2AX was also assessed, this being regarded as a gold standard for the assessment of senescence 20. In sections of ACR; Ki67 negative, γH2AX positive cells were identified with increasing frequency in line with increasing Banff grade. This would strongly suggest that senescent cells are indeed present in increasing number with increased Banff grade, in line with our observations of p21^WAF1/Cip1^. The dual staining of p21^WAF1/Cip1^ and Ki67 was also performed, showing the presence of Ki67 negative p21^WAF1/Cip1^ positive cells in ACR.

An *in vitro* model was established to investigate the possible link between senescence and epithelial de-differentiation. This initially aimed to replicate the findings of Nakanuma and coworkers 8,14, before evaluating the SASP in relation to epithelial plasticity. Transient increases in p21^WAF1/Cip1^ expression by both immortalized and primary BEC were observed after oxidative stress. Staining for further antigens also revealed that immortalized BEC spontaneously underwent de-differentiation following peroxide exposure. This is consistent with observations reported previously for respiratory epithelium 26,27. The phenotype of the primary and immortalized BEC differed in that primary BEC retained their CK19 expression more than immortalized BEC and also retained a more epithelial morphology at respective time points. This may be due to the immortalization process or the presence of a collagen rich scaffold 28, therefore caution must be taken in extrapolating the proteomic results from the *in vitro* to *in vivo* environments.

Several groups have studied factors that may drive epithelial de-differentiation downstream of TGF-β, including Sonic Hedgehog 29–31 and Jagged/Notch 32 (e.g.). However, the factor or factors driving oxidative stress-induced epithelial de-differentiation have not been defined. While the expression of chemokines and interleukins has been studied in relation to the SASP in BEC, the expression of TGF-β has not 8,33. Passos 34 described a putative pathway in fibroblasts undergoing replicative senescence and expressing p21^WAF1/Cip1^ that would increase the expression of TGF-β receptors and the TGF-β2 isoform. To evaluate this, total TGF-β was quantified from the cell culture medium of two time-course experiments using ELISAs specific for TGF-β1 and TGF-β2. These assays showed a slight decrease in total TGF-β1 expression but a fourfold increase in total TGF-β2. To support that this was indeed the mechanism producing the observed de-differentiation, the ALK-5 (TGF-β type I) receptor was blocked using a specific and nontoxic pharmacological inhibitor SB-505124 35. This inhibitor prevented the observed de-differentiation changes normally produced in response to peroxide. This is consistent with the described differential expression of TGF-β1 and β2 transcripts in rodent and human liver fibrosis 36 with a restriction of TGF-β2 transcripts to the biliary epithelium.

To demonstrate that the expression of p21^WAF1/Cip1^ and TGF-β was due to increased transcription, qPCR of isolates from both immortalized and primary BEC were performed. This showed that there was indeed a transient increase in transcripts for p21^WAF1/Cip1^ and TGF-β2 in both cell types. One of the potential confounding factors of using the immortalized BEC is interruption of the p53 signaling pathway by the SV40 large T antigen, the agent used in immortalizing these cells 37. However, it was shown that up-regulation of both p21^WAF1/Cip1^ and TGF-β2 is independent of any action of SV40 as similar expression profiles were produced by the same stimulus in both immortalized and primary BEC. However, a difference in the kinetics of this up-regulation was observed, with primary BEC showing increases at earlier time points than immortalized BEC. This is likely due to the stabilizing effects of SV40 on p21^WAF1/Cip1^ expression, as this is a downstream effecter of p53 38. Ultimately the profiles of gene expression in these cells are comparable within this system and serve to support the use of immortalized BEC as an appropriate model.

TGF-β is produced initially as a pro-protein which is cleaved by furin-like proteases to yield a small latent complex consisting of dimeric TGF-β which is concealed by two latency associated peptides (LAP), This inactive complex can be activated by interaction with the αvβ6 integrin 39–41 which can bind an RGD motif on the LAP leading to a conformational change. The current study showed that resting BEC did not express the β6 integrin, but this was induced following exposure to oxidative stress potentially allowing these cells to activate latent TGF-β.

It has already been shown that expression of markers of proliferation in BEC correlates with Banff grade 25,42. p21^WAF1/Cip1^ can be up-regulated as a consequence of cell cycle regulation, replicative senescence (associated with telomere shortening) or as a result of oxidative stress and other cell damage (e.g. p53 mediated) pathways 22. Acute injurious stimuli can, therefore, lead to senescence in the biliary epithelium, which causes an up-regulation in p21^WAF1/Cip1^. This triggers chemokine and TGF-β2 production, which may drive disease progression. *In vitro* this is observed as de-differentiation in response to TGF-β2. This response is also seen in primary BEC indicating that this is highly likely to model the *in vivo* response of the biliary epithelial compartment to injury. The presence of senescent BEC in ACR may suggest a link between acute and chronic rejection. Senescent BEC are a well-described feature of chronic ductopaenic rejection 43 and as the number of senescent cells correlates with Banff grade 44, so does the probability of future chronic rejection. Chronic rejection has changed, most likely modified by newer anti-rejection agents, the underlying biology of which is not currently understood. Expression of TGF-β subtypes by the biliary epithelium also has the potential to modify the immune system, with a likely increase in regulatory T cells as well as a promotion of wound healing/fibrosis.

We have provided observational evidence of intermediate ‘plastic’ cell types in the bile ducts of human liver biopsies undergoing acute rejection. As a marker of plasticity, it must be remembered that S100A4 is also present in certain normal cell types including macrophages 45 and in the cells of the peribiliary glands 46. Expression of S100A4 has been clearly shown in BEC in the finer branches of the biliary tree and anatomically distinct from the peribiliary glands which are preferentially associated with the intrahepatic large bile ducts. In replicating these findings *in vitro*, we have observed de-differentiation, driven by TGF-β2. Interestingly the response of the immortalized BEC to oxidative stress was to adopt a CK19-, ZO-1-, S100A4+, αSMA+ phenotype and an irregular, stellate morphology. These cells therefore exhibited some characteristics of myofibroblasts and activated hepatic stellate cells (HSC) 15. Any potential role for these stellate-like biliary epithelial cells *in vivo* is however uncertain.

## Abbreviations

ACR: acute cellular rejection
aSMA: alpha smooth muscle actin
BEC: biliary epithelial cells
cDNA: complementary DNA
DAPI: 4′,6-diamidino-2-phenylindole
DMEM: Dulbecco modified Eagle medium
ECL: enhanced chemiluminescence
ECM: extracellular matrix
FITC: fluorescein isothiocyanate
GAPDH: glyceraldehyde-3-phosphate
H_2_O_2_: hydrogen peroxide
HRP: horseradish peroxidase
HSC: hepatic stellate cells
LAP: latency associated peptides
LSCM: laser-scanning confocal microscope
PAGE: polyacrylamide gel electrophoresis
PCR: polymerase chain reaction
ROS: reactive oxygen species
SASP: senescence associated secretory phenotype
SDS: sodium-dodecyl sulphate
TBS: tris-buffered saline

## Supporting Information

**Figure S1: Expression of CK19 and E-Cadherin antigens by H69 biliary epithelial cells.** For both antigens confocal microscopy images are present from control and 48 and 72 h post hydrogen peroxide exposure; alongside densitometric analysis. Panels A–D show CK19 expression, this is significant at p = 0.0273. Panels E–H show E-Cadherin expression, significant at p = 0.039.

**Figure S2: Hydrogen peroxide induced the expression of beta6 integrin on H69 cells.** To ascertain what may be causing the activation of TGF-beta within the H69 treated cells, staining for beta6 containing integrins was performed. This showed a significant up regulation at 48 h post peroxide exposure.

**Figure S3: Autofluorescence of ACR biopsies.** There is clear evidence of lipofuscin granules (white arrows) presence within BEC and hepatocytes, enhancing the data demonstrating senescence marker expression within these cells.
