# Supplementary figures and images for: Biliary Epithelial Senescence and Plasticity in Acute Cellular Rejection
Source: Am J Transplant. 2013 Jun 10;13(7):1688–702. doi: 10.1111/ajt.12271 (PMC3746108; doi:10.1111/ajt.12271)

Supplementary figure 1

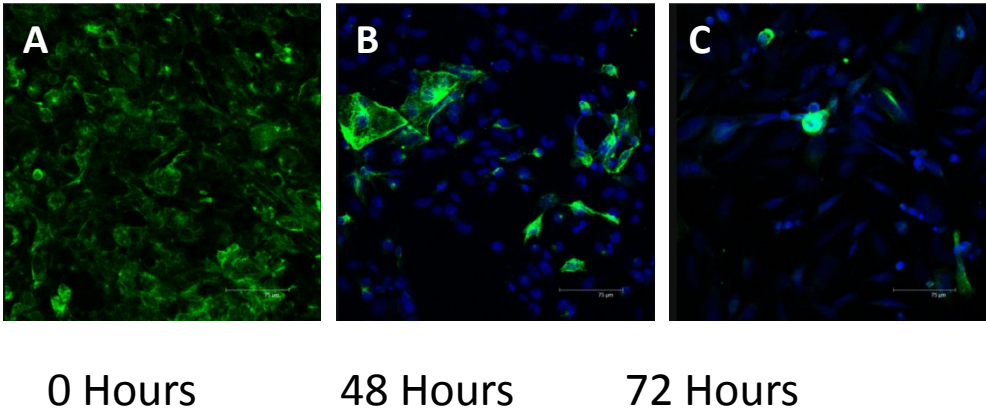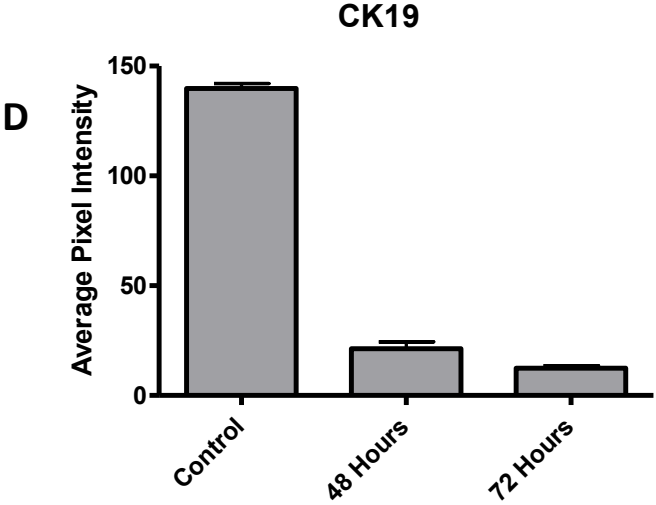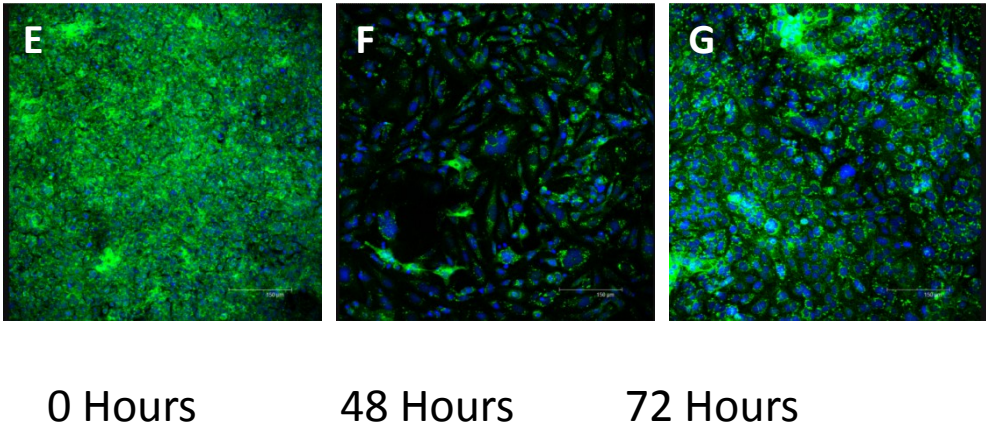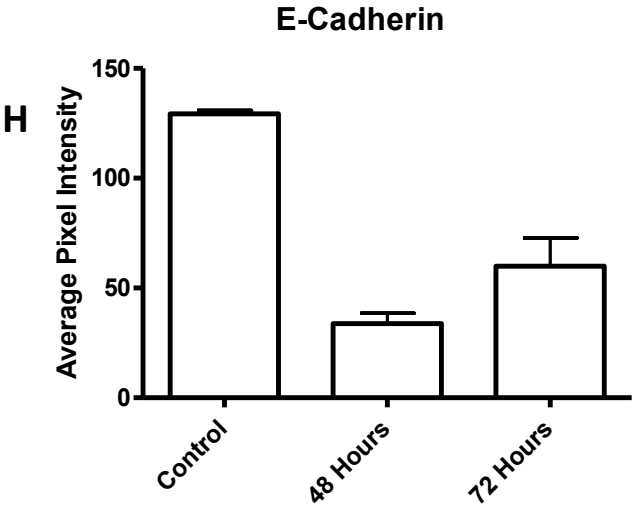

## Supplementary figure 2

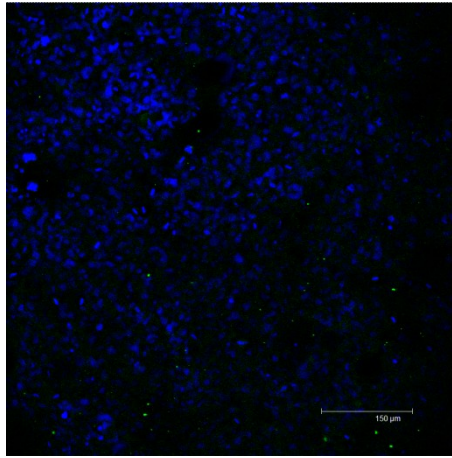

0 Hours

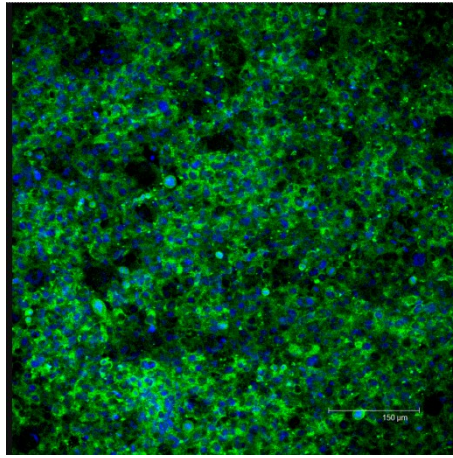

48 Hours

Supplementary figure 3

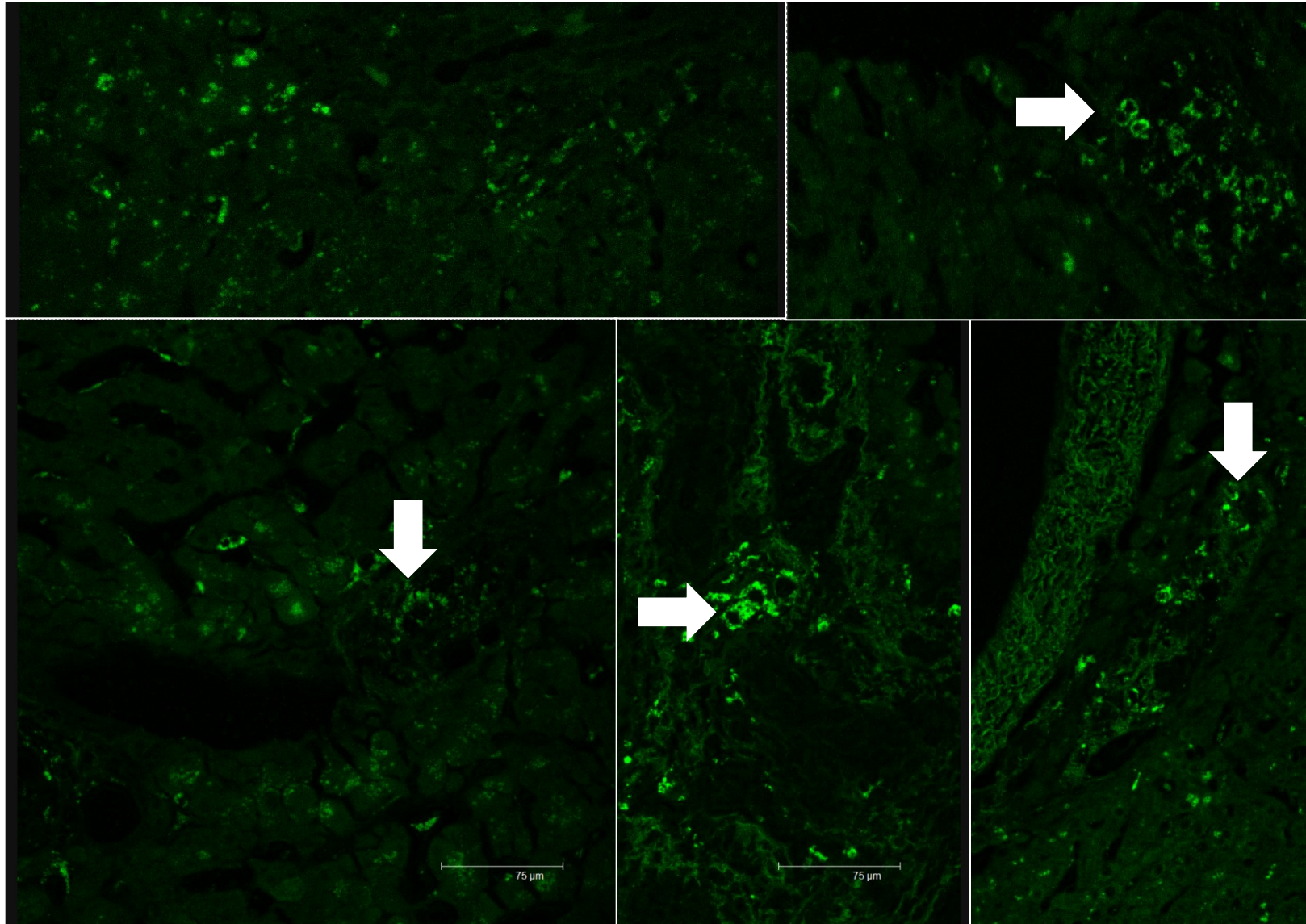

Supplement: Supplementary file 1 [file ajt0013-1688-SD1.pdf]
