# Supplementary material for: Biliary Epithelial Senescence and Plasticity in Acute Cellular Rejection
Source: Am J Transplant. 2013 Jun 10;13(7):1688–702. doi: 10.1111/ajt.12271 (PMC3746108; doi:10.1111/ajt.12271)
Supplement: Supplementary file 2 [file ajt0013-1688-SD2.docx]

# Materials and methods

### Ethics

Ethical approval was obtained from a local regional ethics committee for the use of patient samples. Samples represented excess liver tissue that had been taken for diagnostic purposes; each sample was identified by means of a number allocated by the Cellular Pathology department. Approval reference REC 06/Q0905/150 Amendment 1; date of amendment 09/01/2007 by the Newcastle and North Tyneside Local Research Ethics Committee 1

### Immunohistochemical (IHC) triple staining of human liver biopsies

Paraffin embedded tissue sections were de-waxed by incubation in xylene for 10 minutes and rehydrated by 1 minute washes in 100% and 95% ethanol. To reduce endogenous peroxide activity, sections were incubated in 0.2% hydrogen peroxide in methanol for 10 minutes at room temperature (RT). Antigen retrieval was achieved by pressure cooking (heat-induced antigen retrieval) for 1min in EDTA buffer pH 8 – 9. Several biotinylated secondary antibodies were used; endogenous biotin in the tissue was blocked using a biotin-avidin kit (Vector). Following washing in Tris-buffered saline (TBS pH7.6), sections were incubated in 20% normal swine serum (in TBS) for 1 hour at RT; to reduce non-specific antibody binding. Primary antibody was then added (anti-p21^WAF1/Cip1^ (Santa Cruz) 1:50 or anti-CD3 (DAKO) 1:100) in 20% normal swine serum at 4˚C overnight in a humidified chamber. After washing in TBS, biotinylated goat anti-mouse IgG (Vector, 1:200) in 20% normal swine serum was added for 1 hour at 22˚C. Sections were then washed in TBS and developed with the Vector ABC (peroxide) kit according to the manufacturer’s instructions. The first stage was developed with Nickel 3,3’ diaminobenzidine tetrahydochloride (NiDAB), giving a black colour. Following washing in running tap water then TBS the samples were incubated with a mix of anti-Cytokeratin 19 (DAKO; 1:1000) and anti S100A4 (BD, 1:200) in 20% normal swine serum at 4˚C overnight in a humidified chamber. A further wash with TBS followed by incubation with peroxidase conjugated horse anti-mouse IgG (Vector; 1:100) in 20% normal swine serum for 1 hour at 22˚C. This was then developed with 3, 3’ diaminobenzidine tetrahydochloride (DAB) producing a brown colour where CK19 was detected. A further secondary antibody was subsequently added; biotinylated goat anti-rabbit IgG (Vector; 1:200) in 20% normal swine serum for 1 hour at RT. This was developed with the Vector ABC-AP kit according to the manufacturer’s instructions. The final development was with Vector red in Tris-HCl buffer, pH 8.2-8.4, for 10 min. This gave a red colour where the presence of S100A4 was seen. A light counterstain was added with Mayer’s haematoxylin before dehydration in an ethanol series (70%, 90%, 95% and 100%) and mounting with DPX.

### Analysis

The cases represented 9 time zero biopsies (tissue taken at the point of reperfusion of a transplanted liver) as control tissue and 25 biopsies of transplanted liver with varying grades of acute rejection (mild, moderate and severe). The sections obtained were stained with one set of three antibodies; either p21^WAF1/Cip1^, CK19 and S100A4 or CD3, CK19 and S100A4. Complete triple labelled sections were scanned using an Aperio slide scanner at x20 and remotely accessed files were analyzed. Each biopsy was assessed by two observers (JGB and HR) and scored for number of portal tracts, number of bile duct radicles, maximum number of bile ducts per portal tract, number of p21^WAF1/Cip1^ positive BEC, number of S100A4 BEC, number of dual stained BEC (S100A4 and p21^WAF1/Cip1^) and number of p21^WAF1/Cip1^ and S100A4 BEC adjacent to one another. There was good agreement between the observers, any discrepancies were noted and a consensus opinion reached. As a number of immune cells stain positive for S100A4, there was a chance that infiltrating macrophages or T cells could be mistaken for BEC. This was usually straightforward to assess morphologically. Serial CD3 stained sections allowed some assessment of T cell mediated EMT.

### Cell culture

The H69 cell line was created by Grubman [9] from human intrahepatic BEC. These cells exhibit characteristics of normal human biliary epithelium, with expression of CK7 and 19. The H69 cells were cultured in 25cm^2^ or 75cm^2^ flasks in a mix of Dulbecco’s Modified Eagle’s medium and Nutrient Mixture F12 Ham (3:1) (Sigma) supplemented with: 1.8 x 10^-4^M adenine (24.3mg/l) (Sigma), 2 x 10^-9^M triiodothyronine (1.345μg/l) (Sigma), 5.5 x 10^-6^M epinephrine (1.0mg/l) (Sigma), ITS-X supplement (10mg/l insulin, 5.5g/l transferring, 2.0g/l ethanolamine, 6.7μg/l sodium selenite) (Gibco, Invitrogen), 1μM hydrocortisone solution (362.46μg/l) (Sigma), 10% Heat inactivated FCS (Sigma) and PenStrep (100U/ml of penicillin and streptomycin) (Sigma)

Primary biliary epithelial cells were isolated from human explanted livers as described and were cultured on plasticware coated with rat tail collagen. Culture medium composed: DMEM (Invitrogen), Hams F12 (Sigma-Aldrich), 10% human serum (HD Supplies), EGF (10ng/ml, Peprotech), HGF (10ng/ml, Peprotech), hydrocortisone (2mg/ml Sigma-Aldrich), Insulin (0.124U/ml, Sigma-Aldrich) tri-iodo-thyronine (2nM/ml Sigma-Aldrich), cholera toxin (10ng/ml Sigma-Aldrich), L-glutamine (2mM Gibco), Penicillin (100U/ml Gibco), streptomycin (100ug/ml Gibco).

### Immunofluorescence and densitometry

For immunofluorescence experiments, H69 BEC were seeded on glass 8-chamber slides (Becton Dickinson, UK) at 50,000 cells per chamber with 250µl of culture medium. Cells were cultured for 24-48h until a confluent monolayer was formed. Following optimisation, cells were subjected to oxidative stress by the addition 200µM hydrogen peroxide (final concentration) for 2 hours followed by rinsing in PBS and addition of fresh culture medium. After a preset time (24, 48, 72, 96 or 120 Hours) the cells were rinsed with PBS, fixed with 4% phosphate-buffered paraformaldehyde (30min at 22ºC) and permeabilised with 0.1% Triton X-100 in PBS (15min at 22°C). Cells were then incubated (at 22ºC) with 5% BSA in PBS for 90 minutes to minimize non-specific antibody binding. At this stage the chamber structure was removed from slides.

Primary antibodies specific for S100A4 (DAKO, 1:100), αSMA (Sigma, 1:100), E-cadherin (BD Biosciences, 1:50), ZO-1 (Invitrogen, 1:100), p21^WAF1/Cip1^ (Santa Cruz 1:100), Fibronectin (Sigma 1:100) or Vimentin (DAKO 1:100) were added in 5% BSA for 48 hours at 4°C. For each time point and antigen a no-primary antibody treated preparation and a non-specific isotype/species specific primary antibody preparation were used as negative controls. The cells were then washed with 0.1% Tween-20 in PBS prior to addition of FITC- conjugated anti-mouse or anti-rabbit (DAKO, 1:100) secondary antibody in 5% BSA for 48h at 4^O^C in the dark. Following washing in PBS the slides were stained with DAPI in PBS (at a concentration of 1μg/ml) for 10min at 22^O^C. The slides were then washed in PBS and mounted in fluorescence mounting medium (DAKO). Storage was at 4^O^C.

Visualisation was by immunofluorescence using a Leica TCS SP2 UV laser-scanning confocal microscope (LSCM) for the chromogens: FITC (excitation 488nm, emission 510-535nm) and DAPI (excitation 345nm, emission 450-550nm). Emission levels were set against a control section. Representative z series were taken of each section/specimen and used for densitometric and morphological analysis. Densitometry analysis was by LCS software. All experiments were conducted in triplicate.

### Western blotting

### Protein lysate preparation

H69 growing in 75cm^2^ flasks were rinsed with PBS and drained. 100µl of lysis buffer (Phosphosafe™ Merck KGaA, Darmstadt, Germany) was applied and the cells were scraped off with a cell scraper. The contents of the flasks were then pipetted into centrifuge tubes and then sonnicated for 10 seconds; this was followed by centrifugation at 200g. Supernatant was aliquoted into fresh centrifuge tubes and frozen at -80^O^C. Supernatant Total protein concentration was estimated with a BCA protein assay kit (Pierce, USA). This allowed production of a standard curve for absorbance at 562nm. Extracts were then stored at -20^O^C.

For use in SDS-PAGE; samples were thawed and supplemented with 10% β-mercaptoethanol and 4x NuPAGE sample buffer (Invitrogen). Samples were boiled for 10 minutes, allowed to cool, and then added to SDS-PAGE gels.

### SDS-Polyacrylamide electrophoresis and Western blotting

20µg of total protein lysate per well was separated by 4-12% gradient Sodium-dodecyl sulphate Polyacrylamide gel electrophoresis (SDS-PAGE). Separation was carried out using stable voltage, (20V per gel). Separated proteins were transferred from the gel to Hybond-P nitrocellulose membrane (Amersham Pharmacia Biotech, UK) overnight at 50mA. Non-specific antibody binding sites on the membrane were blocked with 5% skimmed milk in TBS for 90 minutes. Primary antibodies were diluted in 5% milk/TBS to optimal concentrations. The membranes were incubated with primary antibodies overnight at 4^O^C and washed several times with TTBS (TBS supplemented with 0.1% Tween-20). Membranes were then incubated with secondary antibodies conjugated with HRP (Horse Radish Peroxidase) for 1h at RT. After washing with TTBS, the membrane was developed using Pierce Pico Chemiluminescence kit (Pierce). Visualisation was by use of G-Box CCD camera ().

Following development, membranes were washed and stripped of antibodies by incubation with stripping buffer (62.5M Tris, 2% SDS, 100mM β-mercaptoethanol, pH 6.8) at 60^O^C for 30 minutes. Membranes remained in the cooling buffer with agitation for a further 30 minutes. Membranes were washed in TTBS, re-blocked in 5% skimmed milk for 90 minutes and incubated overnight at 4^O^C with anti-β-tubulin antibody (Sigma 1:5000) in 5% skimmed milk and TBS. Secondary antibody was anti-rabbit HRP conjugated antibodies (Sigma 1:5000) with development as for primary antibodies. The intensity of β-tubulin was used to validate equal loading.

### ELISAs

ELISA kits specific for the TGF-β1 and TGF-β2 isoforms were purchased from R&D Systems (UK). Cell culture medium from two separate time courses was retained for analysis. This was first treated with acid (50µl 6M HCl per ml medium for 10 minutes followed by neutralisation with 50µl per ml of 6M NaOH) in order to assess the total rather than the active amount of TGF-β. The kits were then used in accordance with the manufacturer’s instructions.

### Real-time Quantitative Polymerase Chain Reaction (qPCR)

All experiments carried out for qPCR including RNA isolation and cDNA synthesis were done in an RNase free environment to minimise the risk of contamination. RNA isolation was performed according to the method developed by Chomczynski and Sacchi. The method utilises TRIzol reagent (Sigma) a mono-phasic solution of phenol and isothiocyanate to extract total RNA from cells. Cells grown in T75cm2 flasks were re-suspended in 1ml TRIzol RT for 5min to allow TRIzol to disrupt and dissolve cellular components whilst maintaining RNA integrity. The addition of chloroform (200 μl;10mins; RT) followed by centrifugation (12000xg; 15min; 4^O^C) causes the solution to separate into two phases; a lower (organic) phase containing DNA and protein and the upper aqueous phase containing RNA. This upper phase was transferred into a new eppendorf with isopropanol added(0.5ml; 10min; RT) to precipitate the RNA followed by centrifugation (12000xg;10min;4^O^C). The supernatant was removed and the RNA pellet washed with ethanol (1ml;75%) and centrifuged (7500xg; 4min; 4^O^C). The supernatant is again removed and the pellet air dried before re-suspended in 50μL of RNase-free water. The RNA is quantified and quality assessed using a nanodrop spectrophotometer (NanoDrop ND-1000, Thermo scientific, Wilmington USA.),

### First Strand cDNA synthesis

Isolated RNA is reverse transcribed to cDNA using an AffinityScript Multi Temperate cDNA synthesis kit (Agilent technologies). The reverse transcriptase enzyme utilised in the kit is a genetically engineered version of MMLV reverse transcriptase that has improved specific activity over a broad range of temperature from 42^O^C to 55^O^C. The kit can deliver high yields of cDNA from small amounts of RNA (1ng-5ug). In this project, where possible, 5μg of RNA was used per reaction and 3μl of random hexamers were used instead of oligo-dt primers to ensure correct synthesis. The subsequent steps were performed as per manufacturer’s instructions. cDNA was stored at -20^O^C until PCR was performed.

### Real-time Quantitative Polymerase Chain Reaction

qPCR was performed to quantify altered gene expression by amplifying a targeted cDNA molecule. qPCR utilises the same basic method as traditional PCR but qPCR uses fluorogenic probes to allow monitoring of the reaction in ‘real-time’ throughout all three phases; exponential, linear and plateau. Traditional PCR measures only during the plateau phase, with the product visualised as a band on agarose gel detecting only the presence of the target gene, however, qPCR measures during all phases thus allowing for accurate quantitation of target sequence levels, Figure 7 &Table 3.

The qPCR experiments in this project utilised TaqMan (Applied Biosystems) chemistry and SYBRgreen chemistry in the gene array described below. TaqMan uses a fluorogenic probe to guarantee specific hybridisation between probe and target gene sequence. The probe is designed with a reporter high-energy dye at the 5’ end and low energy quencher dye at the 3’ end, Figure 8

qPCR was performed in a MicroAmp Optical 96 well plate (Applied Biosystems) on the StepOnePlus real-time PCR machine (Applied Biosystems)with each well containing 1ul TaqMan primer-probe, 1ul cDNA, 10ul Mastermix and 8ul RNase free water. The primers used were exon-spanning minimising problems associated with genomic DNA contamination. The mastermix used contained ROX, a fluorescent dye that provides an internal reference for the reporter dye to be normalised against therefore minimising complications associated with inter-well error. For each primer triplicate technical replicates were performed and a negative control was included that contained no template. To quantify the relative gene expression a gene of interest cycle threshold (Ct) value is compared against a housekeeping gene Ct e.g. GAPDH by the formula as described by Yuan *et al*(55) giving rise to the corresponding ΔΔCt and fold change values

### Statistical analysis

Statistical analysis was performed using Prism software version 3.0 (Graph Pad Software, San Diego, USA). Parametric data was assessed by Student’s t-test or one way ANOVA, depending on the number of groups and parameters to compare. Statistical significance was defined as a p-value of 0.05 or less. All error bars represent the standard error of the mean (SEM). If a significant result was obtained then post hoc t tests were performed using Bonferroni correction (as the results were not all obtained at the same time). When analysing the triple stained acute rejection biopsies; the spread of data and proximity to zero of a significant proportion of the data (skewing of distribution) prevented the data being considered as normally distributed. Therefore the Kruskall-Wallis non-parametric test was employed as an alternative to a one-way ANOVA.

**Supplementary figure 1:** Expression of CK19 and E-Cadherin antigens by H69 biliary epithelial cells. For both antigens confocal microscopy images are present from control and 48 and 72 hours post hydrogen peroxide exposure; alongside densitometric analysis. Panels A-D show CK19 expression, this is significant at p = 0.0273. Panels E-H show E-Cadherin expression, significant at p = 0.039.

**Supplementary figure 2:** Hydrogen peroxide induced the expression of beta6 integrin on H69 cells. To ascertain what may be causing the activation of TGF-beta within the H69 treated cells, staining for beta6 containing integrins was performed. This showed a significant up regulation at 48 hours post peroxide exposure

**Supplementary Figure 3:** Autofluorescence of ACR biopsies. There is clear evidence of lipofuscin granules (white arrows) presence within BEC and hepatocytes, enhancing the data demonstrating senescence marker expression within these cells.
